# Letter to the editor: Pre-exposure prophylaxis for HIV in Europe: The need for resistance surveillance

**DOI:** 10.2807/1560-7917.ES.2017.22.11.30483

**Published:** 2017-03-16

**Authors:** Carla van Tienen, David van de Vijver, Teymur Noori, Anders Sönnerborg, Charles Boucher

**Affiliations:** 1 Department of Viroscience, Erasmus Medical Center Rotterdam, The Netherlands; 2 European Centre for Disease Prevention and Control, Stockholm, Sweden; 3 Unit of Infectious Diseases and Dermatology, Karolinska Institute, Stockholm, Sweden; 4 The European Society for translational Antiviral Research, Utrecht, The Netherlands

**Keywords:** HIV, resistance, Europe, PrEP, prophylaxis, surveillance

**To the editor:** In a recent paper by Hauser et al. in this journal, a prevalence of 10.8% of transmitted drug-resistant viruses was reported among newly diagnosed HIV cases in Germany in 2013 and 2014 [1]. The authors conclude that genotypic resistance testing remains important for treatment as well as HIV prevention. We comment on the use of pre-exposure prophylaxis (PrEP) in relation to drug resistance in HIV infections and the need for European surveillance of drug resistance.

PrEP with tenofovir and emtricitabine prevents new HIV infections in persons at high risk of acquiring HIV [2]. In 2016, the European Commission approved emtricitabine/tenofovir disoproxil once per day for PrEP. France and Norway are the only two countries in Europe fully reimbursing PrEP but many more are considering implementing PrEP pilot projects in 2017 and 2018 [3]. PrEP is cost effective with the current drug prices [4,5] and a generic version of tenofovir and emtricitabine is expected in 2017 or 2018, which may reduce the costs and lead to more widespread use of PrEP in Europe.

PrEP use also poses some challenges as the included drugs are part of the recommended first and second line regimens to treat HIV-infected individuals. The resistance patterns that develop against either drug in a situation of therapy failure are well known: the primary mutation selected by tenofovir that causes a diminished treatment response is the K65R amino acid substitution in the reverse transcriptase. In addition, the presence of multiple thymidine-associated mutations (TAMs) selected by zidovudine, a previously frequently used drug in HIV treatment, can affect the effect of tenofovir on the virus. In individuals failing emtricitabine (or the commonly used lamivudine)-containing regimens, the amino acid changes M184I/V are frequently seen [6]. Viruses with these mutations can be transmitted, resulting in the failure of tenofovir/emtricitabine-based PrEP [7,8].

The use of PrEP by individuals infected with HIV but unaware of this can lead to the generation of resistant viruses in these individuals. Transmission to, or selection in, an HIV-positive person on PrEP carries the risk of forward transmission of these resistant virus to other individuals (both on and off PrEP).

Therefore, we recommend surveillance on national level as well European level. As mentioned by Hauser et al., Germany has a mandatory notification system of new HIV diagnoses, but this is not the case in all European countries [1]. In addition, baseline genotypic resistance testing is not routinely performed in all countries. We recommend the surveillance network *Strategy to Control Spread of HIV Drug Resistance* (SPREAD) to collect these data [9]. SPREAD is organised in 28 countries by the European Society of Antiviral Research (ESAR) and monitors drug-resistant viruses in newly diagnosed individuals [10]. SPREAD can add the use of PrEP in the baseline questionnaire and install a registry within the existing SPREAD database, collecting data on selection of resistant viruses and treatment and/or prophylaxis failure due to PrEP use. In this way, we hope that outbreaks of PrEP-resistant viruses will be identified in a timely manner.

In conclusion, as PrEP for HIV prevention is expected to be rolled out in European countries in the near future, and considering the informal use of PrEP in the community, we suggest including variables on PrEP use in the European surveillance SPREAD programme, increasing the proportion of baseline resistance testing in newly diagnosed HIV infections and installing a registry on the selection of resistant viruses and failure of PrEP within the existing SPREAD database.

## Note

Disclaimer: This paper is the view of the authors and not necessarily that of the agencies that they represent.
